# Supplementary material for: Gliding on Ice in Search of Accurate and Cost-Effective Computational Methods for Astrochemistry on Grains: The Puzzling Case of the HCN Isomerization
Source: J Chem Theory Comput. 2022 Apr 21;18(5):3111–21. doi: 10.1021/acs.jctc.1c01252 (PMC9097295; doi:10.1021/acs.jctc.1c01252)
Supplement: Supplementary file 1 — ct1c01252_si_001.pdf [file ct1c01252_si_001.pdf]

Supporting Information

Gliding on ice in search of accurate and cost-effective computational methods for astrochemistry on grains: the puzzling case of the HCN isomerization

Carmen Baiano, Jacopo Lupi, Vincenzo Barone,* and Nicola Tasinato*

Scuola Normale Superiore, Piazza dei Cavalieri 7, I-56126, Pisa, Italy

E-mail: vincenzo.barone@sns.it; nicola.tasinato@sns.it

List of supporting information

Table S1. Main structural parameters for the water dimer obtained at the ChS and jun-ChS levels and comparison to CCSD(T)-F12b/CBS+fT+fQ+CV+REL+DBOC values

Table S2. Mean Absolute Errors and Relative Errors for bond lengths, angles and dihedrals of the HCN···$(H_2O)_2$ system.

Figure S1. Mean Absolute Errors (MAE) and Relative Errors (RE) for bond lengths, valence and dihedral angles.

Table S3. Contributions to jun-ChS electronic energies.

Table S4. Absolute Errors and Mean Absolute Errors of formation energies computed at the jun-ChS level on top of different optimized geometries with respect to full jun-ChS results.

Figure S2. Structure of the HCN@(H_2O) system showing molecules frozen during the optimization.

Table S5. Relative ground state electronic energies for the stationary points on the HCN⇌HNC isomerization PES with respect to isolated HCN and $(H_2O)_n$ for $n = 2, 20$.

Table S7. Cartesian coordinates of the stationary points on the HCN⇌HNC isomerization PES on a $(H_2O)_n$ cluster for n = 2, 3, 4 optimized at the DSDPBEP86-D3/jul-cc-pVTZ level of theory.

Table S7. Cartesian coordinates of the stationary points on HCN⇌HNC isomerization PES catalyzed by the $(H_2O)_{20}$ cluster optimized at the DSDPBEP86-D3/jul-cc-pVTZ:PW6B95-D3/jul-cc-pVDZ level of theory.

Table S1 Main structural parameters for the water dimer obtained at the ChS and jun-ChS levels and comparison to CCSD(T)-F12b/CBS+fT+fQ+CV+REL+DBOC values of ref.[1] θ_a and θ_d are the H-O-H valence angles for the acceptor and the donor respectively, α gives a measure of the deviation from a linear hydrogen bond and β gives the orientation of the C_2 axis of the acceptor with respect to the O-O axis. Bond lengths in Å and angles in degrees.

	**ChS**	**jun-ChS**	**Ref. value**[1]
**r(O-O)**	2.9049	2.9058	2.9092
$\boldsymbol{\theta_a}$	104.78	104.77	104.95
$\boldsymbol{\theta_d}$	104.87	104.84	104.85
$\boldsymbol{\alpha}$	4.91	5.91	5.69
$\boldsymbol{\beta}$	126	127	123.46

Table S2 MAE and RE for bond lengths (**r**), angles (**α**) and dihedrals (**ϕ**) of the HCN–$(H_2O)_2$ system. Averaged values over all the species along the PES are collected. Last column collect total RE obtained averaging over all the structural parameters. MAE for bond lengths in Å and in degrees for angles and dihedrals. RE are in %.

		MAE			RE			
		**r**	**α**	**ϕ**	**r**	**α**	**ϕ**	**Total**
**B3LYP-D3**	**Jun-DZ**	0.028	1.72	2.21	1.78	1.31	2.43	1.02
	**Jul-DZ**	0.023	1.45	3.56	1.47	1.23	3.65	0.98
	**Aug-DZ**	0.021	1.24	2.53	1.36	1.08	2.61	0.88
	**Jun-TZ**	0.013	1.07	2.69	0.87	0.89	3.08	0.69
	**Jul-TZ**	0.013	1.03	2.44	0.86	0.86	2.69	0.65
	**Aug-TZ**	0.013	1.09	2.51	0.89	0.92	2.82	0.69
**BHLYP-D3**	**Jun-DZ**	0.092	7.34	9.21	3.50	6.17	10.47	4.82
	**Jul-DZ**	0.016	1.64	2.52	0.87	1.39	2.38	0.85
	**Aug-DZ**	0.014	1.46	1.64	0.79	1.25	1.81	0.76
	**Jun-TZ**	0.014	1.28	1.59	0.96	1.07	1.65	0.80
	**Jul-TZ**	0.014	1.34	1.54	0.95	1.10	1.82	0.83
	**Aug-TZ**	0.015	1.34	1.48	0.97	1.11	1.72	0.83
**ωB97X-D**	**Jun-DZ**	0.030	2.36	4.01	1.65	1.90	3.28	1.06
	**Jul-DZ**	0.023	1.82	5.01	1.25	1.57	4.86	0.91
	**Aug-DZ**	0.021	1.70	5.15	1.14	1.48	4.89	0.92
	**Jun-TZ**	0.017	1.63	5.06	0.88	1.37	5.23	0.81
	**Jul-TZ**	0.017	1.59	4.87	0.88	1.34	4.95	0.78
	**Aug-TZ**	0.018	1.62	5.00	0.93	1.38	5.17	0.81
**PW6B95-D3**	**Jun-DZ**	0.022	2.03	2.47	1.23	1.50	2.48	1.00
	**Jul-DZ**	0.015	1.34	3.11	0.80	1.09	3.05	0.74
	**Aug-DZ**	0.013	1.05	1.96	0.73	0.90	1.65	0.60
	**Jun-TZ**	0.011	0.96	2.56	0.55	0.79	2.59	0.55

	**Jul-TZ**	0.010	0.94	2.31	0.53	0.76	2.29	0.52
	**Aug-TZ**	0.010	0.95	2.34	0.52	0.78	2.36	0.53
**BMK-D3**	**Jun-DZ**	0.015	1.87	3.32	1.00	1.36	2.71	0.75
	**Jul-DZ**	0.013	1.67	4.09	0.84	1.38	3.84	0.77
	**Aug-DZ**	0.012	1.60	2.84	0.77	1.34	2.47	0.69
	**Jun-TZ**	0.008	1.03	2.32	0.52	0.84	2.04	0.57
	**Jul-TZ**	0.008	1.00	2.00	0.52	0.81	1.61	0.54
	**Aug-TZ**	0.008	1.03	2.02	0.51	0.84	1.68	0.56
**M06-2X**	**Jun-DZ**	0.014	1.95	2.65	1.05	1.37	2.99	1.09
	**Jul-DZ**	0.013	1.16	2.96	0.91	0.96	2.72	0.85
	**Aug-DZ**	0.014	1.18	2.62	0.90	0.96	2.74	0.84
	**Jun-TZ**	0.012	1.05	2.36	0.72	0.82	2.06	0.70
	**Jul-TZ**	0.012	1.00	1.90	0.74	0.79	1.39	0.65
	**Aug-TZ**	0.012	1.04	1.86	0.75	0.83	1.41	0.66
**MN15**	**Jun-DZ**	0.017	2.15	3.08	1.18	1.59	2.62	1.15
	**Jul-DZ**	0.013	1.49	3.70	0.94	1.17	3.23	0.95
	**Aug-DZ**	0.010	1.20	2.80	0.82	0.99	2.87	0.83
	**Jun-TZ**	0.009	1.03	3.13	0.63	0.82	2.48	0.68
	**Jul-TZ**	0.008	1.04	2.69	0.60	0.82	1.99	0.65
	**Aug-TZ**	0.008	1.01	2.60	0.58	0.80	1.93	0.65
**B2PLYP-D3**	**Jun-DZ**	0.025	1.88	2.36	1.58	1.39	2.89	1.10
	**Jul-DZ**	0.020	1.23	2.71	1.29	1.03	2.62	0.88
	**Aug-DZ**	0.018	1.00	1.74	1.21	0.88	1.65	0.75
	**Jun-TZ**	0.010	0.77	1.65	0.63	0.63	1.77	0.51
	**Jul-TZ**	0.009	0.69	2.13	0.58	0.57	2.72	0.59
	**Aug-TZ**	0.009	0.74	1.50	0.60	0.63	1.60	0.48
**DSD-PBEP86-D3**	**Jun-DZ**	0.021	1.78	2.48	1.44	1.28	3.02	1.07

	**Jul-DZ**	0.017	0.88	2.63	1.18	0.74	2.60	0.76
	**Aug-DZ**	0.016	0.96	2.39	1.15	0.84	2.64	0.81
	**Jun-TZ**	0.008	0.54	1.50	0.60	0.44	1.69	0.43
	**Jul-TZ**	0.007	0.46	1.39	0.57	0.37	1.55	0.41
	**Aug-TZ**	0.007	0.59	1.53	0.58	0.49	1.73	0.44
**rDSD-PBEP86-D3**	**Jun-DZ**	0.023	1.80	2.53	1.49	1.31	2.98	1.11
	**Jul-DZ**	0.017	0.89	2.64	1.15	0.75	2.58	0.77
	**Aug-DZ**	0.016	0.96	2.38	1.12	0.83	2.64	0.80
	**Jun-TZ**	0.008	0.52	1.51	0.58	0.42	1.66	0.43
	**Jul-TZ**	0.007	0.43	1.36	0.54	0.35	1.50	0.40
	**Aug-TZ**	0.007	0.54	1.53	0.55	0.45	1.73	0.42

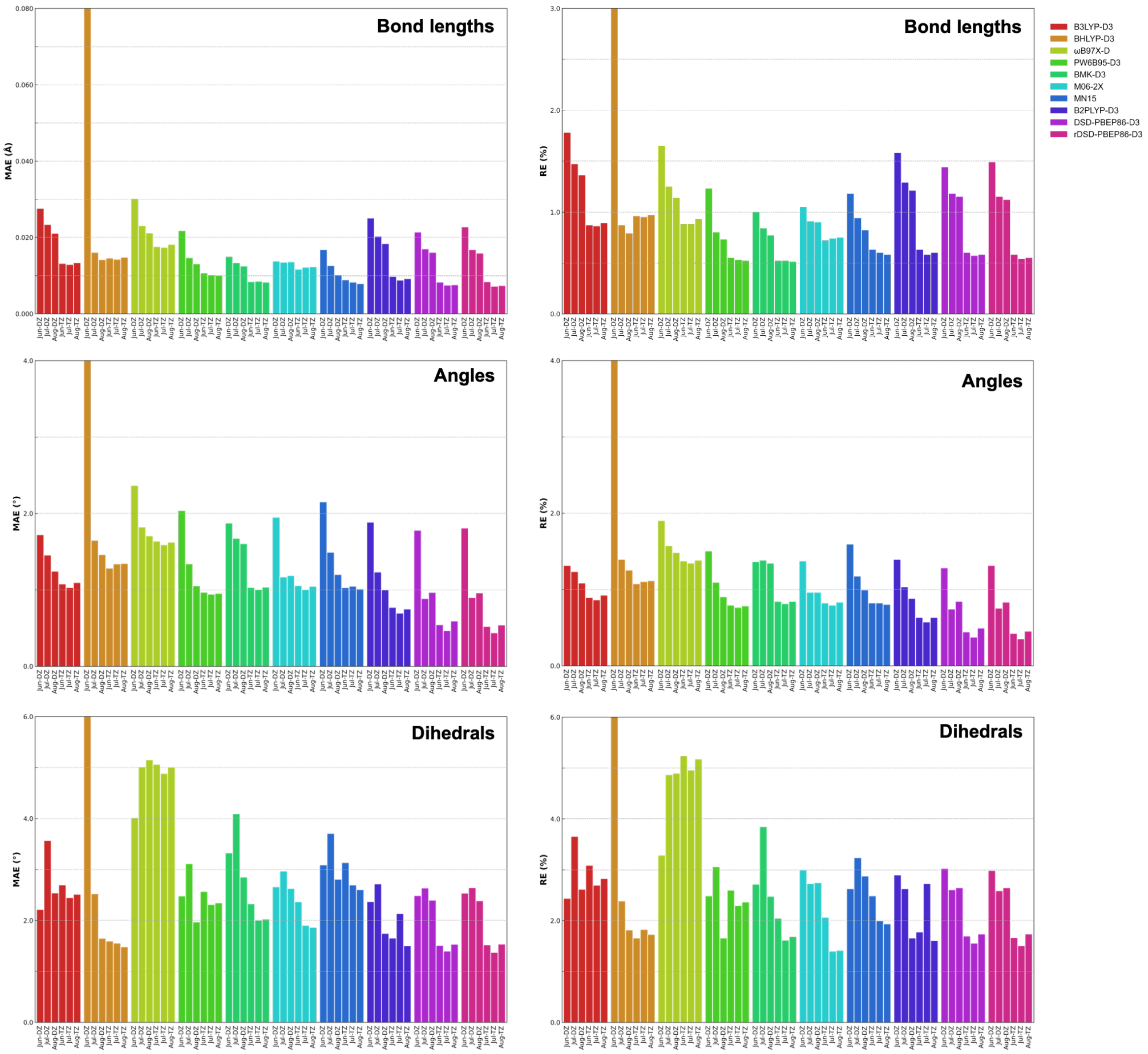

Figure S1 Mean Absolute Errors (MAE) and Relative Errors (RE) for bond lengths, valence and dihedral angles. The values are obtained by averaging absolute and relative errors of structural parameters over all the species involved in the reactive PES for the HCN⇌HNC isomerization assisted by two water molecules.

Table S3 jun-ChS contributions (in Hartree) to energy evaluated on jun-ChS geometries. E(CC) is the CCSD(T) energy computed with the jun-cc-pVTZ basis set; ΔE^{∞}_{MP2} is the difference between the fc-MP2/jun-cc-pVTZ energy and the corresponding extrapolated value estimated by the jun-cc-pVnZ basis sets with n = T and Q. ΔE^{CV}_{MP2} accounts for core-valence correlation and is obtained as difference between ae- and fc- MP2 calculations with cc-pwCVTZ basis set. The final jun-ChS energy is reported in the last row.

	**HCN**	**$(H_2O)_2$**	**HCN–$(H_2O)_2$**	**TS**	**HNC–$(H_2O)_2$**	**HNC**
**E(CC)**	-93.2774	-152.6856	-245.9753	-245.9254	-245.9574	-93.2540
**ΔE^{∞}_{MP2}**	-0.0446	-0.0818	-0.1268	-0.1262	-0.1264	-0.0444
**ΔE^{CV}_{MP2}**	-0.0944	-0.1029	-0.1974	-0.1974	-0.1973	-0.0943
**E(jun-ChS)**	-93.4165	-152.8703	-246.2995	-246.2490	-246.2810	-93.3927

Table S4 AE and MAE of formation energies computed at the jun-ChS level on top of different optimized geometries with respect to full jun-ChS results. The formation energy of each species (in kJ/mol) is calculated with respect to isolated HCN + $(H_2O)_2$. RC stands for reactant complex, i. e. NCH–$(H_2O)_2$, TS for transition state and PC for product complex, i. e. CNH–$(H_2O)_2$.

	**RC**	**TS**	**PC**	**HCN + $(H_2O)_2$**	**MAE**
**PW6B95-D3/jul-DZ**	0.04	0.11	0.20	0.04	0.10
**BHLYP-D3/aug-DZ**	0.01	0.05	0.16	0.39	0.15
**PW6B95-D3/aug-DZ**	0.02	0.15	0.13	0.01	0.08
**BMK-D3/aug-DZ**	0.29	0.12	0.00	0.01	0.11
**M06-2X/aug-DZ**	0.09	0.41	0.34	0.01	0.21
**MN15/aug-DZ**	0.03	0.11	0.57	0.36	0.27
**PW6B95-D3/jul-TZ**	0.07	0.11	0.30	0.38	0.22
**BMK-D3/jul-TZ**	0.05	0.08	0.45	0.61	0.30
**M06-2X/jul-TZ**	0.04	0.23	0.12	0.42	0.20
**MN15/jul-TZ**	0.09	0.07	0.05	0.24	0.11
**DSD-PBEP86-D3/jul-TZ**	0.10	0.09	0.03	0.05	0.07
**rDSD-PBEP86-D3/jul-TZ**	0.12	0.06	0.02	0.05	0.06

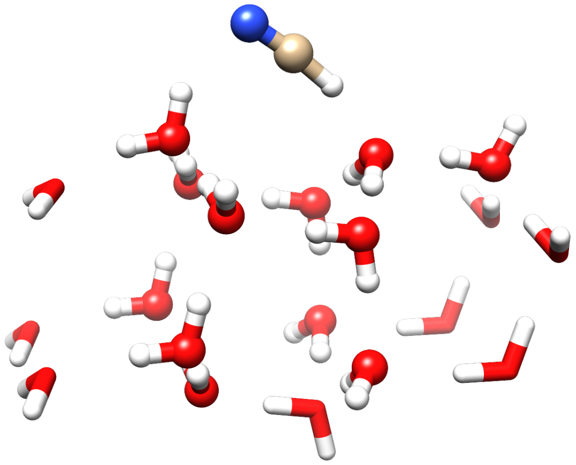

Figure S2 Structure of the HCN@(H_2O) system showing molecules frozen during the optimization. Ball and stick representation used for atoms free to move while tubular representation for molecules kept frozen in order to prevent structural distortion of the cluster.

Table S5 Relative ground state electronic energies for the stationary points on the HCN⇌HNC isomerization PES with respect to isolated HCN and $(H_2O)_n$. The number of water molecules involved in the relay mechanism is explicitly indicated together with the total number of water molecules included in the model system (in parenthesis). ZPVEs are in bold while energy corrected for ZPVEs are in parenthesis. Values in kJ/mol. jun-ChS energy and geometries and ZPVEs at DSD-PBEP86-D3/jul-cc-pVTZ for n=2. jun-ChS energies for the adsorbate and the water molecules involved in the proton relay and PW6B95D3/jul-cc-pVDZ for the remaining water molecules in the cluster for n=4 [20]. PW6B95-D3/jul-cc-pVDZ for PW6B95 results. DSD-PBEP86 results refers to ONIOM(DSD-PBEP86/jul-cc-pVTZ:PW6B95D3/jul-cc-pVDZ), both geometries and ZPVEs at this level.

		**HCN–$(H_2O)_n$**	**TS**	**HNC–$(H_2O)_n$**	**HNC + $(H_2O)_n$**
	n=2 [2]	-33.4	99.3	15.0	62.3
		**6.3**	**1.9**	**7.8**	**-1.3**
**jun-ChS**		(-27.2)	(101.2)	(22.8)	(61.0)
	n = 4 [20]	-61.4	43.4	-18.5	62.3
		**10.6**	**-9.3**	**8.6**	**-2.0**
		(-50.7)	(34.2)	(-9.9)	(60.2)
	n=4 [20]	-64.1	27.6	-27.8	56.7
**PW6B95**		**10.6**	**-9.3**	**8.6**	**-2.0**
		(-53.5)	(18.4)	(-19.2)	(54.7)
	n=4 [20]	-65.4	28.3	-21.6	64.9
**DSD-PBEP86**		**7.9**	**-8.9**	**8.1**	**-1.3**
		(-57.5)	(19.4)	(-13.4)	(63.6)

Table S6 Cartesian coordinates of the stationary points on the HCN⇌HNC isomerization PES on a $(H_2O)_n$ cluster for n = 2, 3, 4 optimized at the DSDPBEP86-D3/jul-cc-pVTZ level of theory.

**HCN···$(H_2O)_2$**
H -2.4172460000 -1.3236170000 -0.3753170000
H -1.4926540000 -0.1512860000 0.0046880000
O -1.5878820000 -1.1174680000 0.0614470000
H 0.2173170000 1.4761330000 -0.1085270000
H -0.8952870000 2.2488950000 0.6011570000
O -0.7411690000 1.5897050000 -0.0809160000
C 1.3176910000 -0.7998110000 0.0131070000
H 0.5103540000 -1.5088020000 0.0288850000

N 2.1148270000 0.0399500000 -0.0105400000
**TS@$(H_2O)_2$**
C -1.2945800000 -0.8862040000 0.0452440000
H 0.3338370000 -1.2836170000 0.1070700000
N -1.6744600000 0.2242890000 -0.0376320000
H -0.3449010000 1.1193430000 -0.1006790000
O 1.3191180000 -0.9595600000 0.0912560000
O 0.6612240000 1.3491600000 -0.0925100000
H 0.8305300000 1.8625930000 0.7062840000
H 1.1046330000 0.2572530000 -0.0053710000
H 1.7218600000 -1.3251680000 -0.7053130000
**HNC···$(H_2O)_2$**
C -2.2892940000 0.4017320000 -0.0124330000
H 0.3174650000 1.5296530000 -0.0990770000
N -1.3931210000 -0.3518240000 0.0118970000
H -0.5499480000 -0.9398710000 0.0254200000
O 1.2690700000 1.3714520000 -0.0833590000
O 1.1166430000 -1.3928350000 0.0944840000
H 1.5599600000 -1.8957890000 -0.5933260000
H 1.4530840000 -0.4808450000 0.0269700000
H 1.6213520000 2.0102850000 0.5423370000
**HCN···$(H_2O)_3$**
O -2.3584400000 0.5238630000 -0.0460450000
H -1.8944140000 -0.3373700000 -0.0302560000
H -3.0921230000 0.4155400000 -0.6550970000
O -0.7934060000 -1.7514890000 0.0645970000
H -0.8862590000 -2.3150170000 0.8367420000

H 0.1662870000 -1.5849630000 -0.0138990000
O 1.9246420000 -1.1641700000 -0.0551470000
H 2.4942560000 -1.4374660000 -0.7788180000
H 2.0268370000 -0.2025640000 0.0038430000
C 0.2842930000 1.7579010000 0.0507490000
H -0.7931660000 1.6577270000 0.0011210000
N 1.4414940000 1.7701540000 0.0892320000
**TS@$(H_2O)_3$**
O -2.1012760000 -0.2913010000 0.0379170000
H -1.2555920000 0.7956740000 0.0165190000
H -2.6039900000 -0.4087480000 0.8500230000
O -0.5665470000 1.6243910000 -0.0630800000
H -0.7096130000 2.0504520000 -0.9152650000
H 0.5149180000 1.3128350000 0.0146840000
O 1.7561250000 0.9443090000 0.0489610000
H 2.1998450000 1.1557290000 0.8764210000
H 1.7168340000 -0.0787420000 -0.0125640000
C 0.0653410000 -1.8276070000 -0.0776250000
H -1.4372350000 -1.0764350000 -0.0136650000
N 1.2109090000 -1.5720440000 -0.0772550000
**HNC···$(H_2O)_3$**
O -2.1812290000 0.6508380000 0.0210110000
H -0.5818510000 1.4812480000 0.0188820000
H -2.8082780000 0.7829560000 0.7371090000
O 0.3017000000 1.8990520000 -0.0250100000
H 0.2527440000 2.5149750000 -0.7603410000
H 1.6418500000 0.7824770000 0.0090730000

O 2.2587060000 0.0165140000 0.0087320000
H 2.8558970000 0.1515870000 0.7485510000
H 1.1438380000 -1.2122110000 -0.0030890000
C -0.8619140000 -2.1038320000 -0.0710360000
H -2.0754540000 -0.3100620000 -0.0492460000
N 0.2441880000 -1.7284580000 -0.0446550000
**HCN···$(H_2O)_4$**
O -2.8436820000 -0.7440310000 -0.0389370000
H -3.5830050000 -0.6505780000 -0.6435400000
H -2.4805420000 0.1586880000 0.0816930000
H 1.1958030000 2.1797050000 -1.2964940000
H 1.7053460000 1.2727840000 -0.1574010000
O 1.0214320000 1.9333040000 -0.3852770000
H -0.6675230000 1.7396320000 0.0638830000
O -1.6156500000 1.6702220000 0.3027840000
H -1.7042870000 2.1484650000 1.1303760000
H 2.3982480000 -0.8863900000 0.2053580000
O 2.8361970000 -0.0261130000 0.3163660000
H 3.7516140000 -0.1581190000 0.0597530000
C -0.1950600000 -1.9590570000 -0.0909090000
H -1.2573540000 -1.7304940000 -0.1327920000
N 0.9465260000 -2.1409150000 -0.0464100000
**TS@$(H_2O)_4$**
O 1.8176240000 -0.7589480000 -1.2502330000
H 2.7630220000 -0.9296150000 -1.3078420000
H 1.0464500000 -1.2976530000 0.0229690000
H -2.2791540000 -1.8623350000 -0.3765390000

H -2.0530740000 -0.3134370000 -0.1386080000
O -1.9098530000 -1.2269600000 0.2418240000
H -0.5166790000 -1.3346420000 0.6023600000
O 0.4968710000 -1.3021280000 0.8765870000
H 0.6638200000 -0.3725560000 1.1918100000
H -1.0244670000 1.4993950000 -0.2833040000
O -1.9563890000 1.2618790000 -0.5212820000
H -2.1235360000 1.6483900000 -1.3837640000
C 1.6206590000 1.4263090000 0.9577060000
H 1.7402660000 0.1775530000 -0.9798640000
N 0.6390540000 1.4909000000 0.3044830000

**HNC···$(H_2O)_4$**
O -2.9134910000 -0.2603150000 -0.3272480000
H -3.7881330000 -0.4487370000 0.0212990000
H -1.8360840000 1.0970350000 0.1226290000
H 1.5413260000 2.3638860000 -1.0133720000
H 2.3984320000 0.3300010000 -0.0403770000
O 1.4584710000 1.7588820000 -0.2731510000
H 0.4988250000 1.7298220000 -0.0664370000
O -1.1933760000 1.7966160000 0.3571760000
H -1.3536570000 1.9869640000 1.2845570000
H 1.4285180000 -1.4920410000 0.1149690000
O 2.7653720000 -0.5620600000 0.1571390000
H 3.4949620000 -0.6988150000 -0.4515920000
C -0.6853020000 -2.1002670000 0.0419490000
H -2.3963510000 -1.0790810000 -0.2119390000
N 0.4554520000 -1.8646310000 0.0967480000

Table S7 Cartesian coordinates of the stationary points on HCN⇌HNC isomerization PES catalyzed by the $(H_2O)_{20}$ cluster optimized at the DSDPBEP86-D3/jul-cc-pVTZ:PW6B95-D3/jul-cc-pVDZ level of theory

$\mathbf{HCN \cdots (H_2O)_{20}}$
H 5.0223470000 0.6152780000 1.3926780000
H 5.0186410000 0.5991620000 3.0286570000
O 5.2779240000 0.0944080000 2.2061930000
H -1.9111380000 1.9471350000 1.6036090000
H -1.8348980000 2.0006570000 3.1507030000
O -1.7156920000 1.4160370000 2.3996800000
H 0.1653830000 0.9696300000 2.2828750000
O 1.1094180000 0.8078430000 2.1577780000
H 1.1991970000 -0.1621710000 2.1672440000
H 4.5199270000 -1.3906580000 2.1901060000
O 4.1402550000 -2.3164700000 2.1799070000
H 3.1352740000 -2.2918190000 2.1780850000
O 1.1494160000 -1.9732040000 2.2230220000
H 0.6759950000 -2.3897110000 2.9450200000
H 0.9487140000 -2.5015840000 1.4261470000
H -2.3589060000 -0.3750370000 2.3360530000
O -2.6377900000 -1.2978890000 2.2442720000
H -3.6008880000 -1.3032660000 2.2955080000
O -5.5380320000 -1.5969230000 2.1649560000
H -5.9101890000 -2.0423080000 2.9837540000
H -5.9064200000 -2.0262330000 1.3359450000
H 5.0324660000 0.6580680000 -3.0031550000

H 5.0286490000 0.6419680000 -1.3671390000

O 5.2879850000 0.1373770000 -2.1895510000

H 5.1655410000 2.5430170000 0.0313410000

O 4.6629020000 1.7269720000 0.0224490000

H 3.7038290000 1.9670060000 0.0214180000

O 2.0453150000 2.1369090000 0.0169970000

H 1.7227370000 1.6244270000 0.7990460000

H 1.7263090000 1.6264310000 -0.7681660000

H -1.7816160000 2.1166320000 -2.9878330000

H -1.8895320000 1.9825250000 -1.4487230000

O -1.7031660000 1.4830340000 -2.2695180000

H 0.1603500000 1.0050480000 -2.2102460000

O 1.1131330000 0.8499970000 -2.1403070000

H 1.2057010000 -0.1216620000 -2.1698560000

H 4.5299660000 -1.3477240000 -2.2059210000

O 4.1503120000 -2.2735520000 -2.2155260000

H 3.1453310000 -2.2488970000 -2.2178220000

O 1.1607940000 -1.9269410000 -2.2632470000

H 0.9559960000 -2.4721380000 -1.4787980000

H 0.6870260000 -2.3247140000 -2.9955280000

H -2.2224330000 -2.1754670000 -0.8082580000

H -2.2230240000 -2.1960950000 0.7529520000

O -2.0435270000 -2.7428290000 -0.0348670000

H -0.5019290000 -3.2108320000 -0.0373410000

O 0.4879280000 -3.3850830000 -0.0369310000

H 0.6773690000 -4.3677840000 -0.0459050000

H -2.1560840000 3.7162930000 0.0579700000

O -2.3832480000 2.7746330000 0.0811710000
H -3.3528650000 2.6797420000 0.0452800000
O -4.9515700000 1.9390070000 0.0027750000
H -5.4141020000 1.6125670000 0.8247850000
H -5.4104170000 1.6290200000 -0.8277360000
H -2.3590370000 -0.3031570000 -2.3144620000
O -2.6319990000 -1.2311800000 -2.2710360000
H -3.5944820000 -1.2408370000 -2.3329900000
O -5.5279770000 -1.5539750000 -2.2307920000
H -5.9001370000 -1.9994150000 -1.4120420000
H -5.8963470000 -1.9833070000 -3.0598430000
C 0.2828100000 4.4290600000 -0.0333840000
H 1.1074610000 3.7075660000 -0.0169380000
N -0.6608130000 5.1018440000 -0.0456510000
**TS@$(H_2O)_{20}$**
H 5.0265310000 0.5452200000 1.3719930000
H 5.0243990000 0.5023880000 3.0075780000
O 5.2673330000 0.0037570000 2.1765170000
H -1.8131370000 1.7251130000 1.5040760000
H -1.8259640000 1.9623530000 3.0461000000
O -1.6354100000 1.2984150000 2.3807060000
H 0.1842190000 0.9593860000 2.3147530000
O 1.1229330000 0.7751720000 2.1488750000
H 1.1924610000 -0.1968860000 2.1648670000
H 4.4646060000 -1.4572860000 2.1371270000
O 4.0570330000 -2.3708920000 2.1124870000
H 3.0531930000 -2.3157370000 2.1126000000

O 1.0776810000 -1.9911820000 2.2040570000

H 0.6083250000 -2.3981010000 2.9343450000

H 0.8414370000 -2.5004300000 1.4058740000

H -2.3579880000 -0.4109460000 2.3579220000

O -2.6946680000 -1.3140000000 2.2391200000

H -3.6568840000 -1.2510280000 2.2730780000

O -5.5949530000 -1.3583940000 2.1256260000

H -5.9795410000 -1.8057370000 2.9375870000

H -5.9772420000 -1.7625520000 1.2902690000

H 5.0327930000 0.6604830000 -3.0224560000

H 5.0303750000 0.6175640000 -1.3868760000

O 5.2734940000 0.1190020000 -2.2180000000

H 5.1520480000 2.5119370000 0.0476240000

O 4.6587140000 1.6903810000 0.0076210000

H 3.7064870000 1.9218980000 0.0257500000

O 1.9825860000 2.0111210000 0.0347930000

H 1.6552760000 1.5542270000 0.8699970000

H 1.3697740000 1.2756690000 -1.1905870000

H -1.5953630000 2.0837570000 -2.7700470000

H -1.7904590000 1.8992670000 -1.0877650000

O -1.4594110000 1.4250110000 -2.0817720000

H -0.1104420000 1.1172370000 -2.0320600000

O 0.9288070000 0.8545390000 -2.0022150000

H 1.0141520000 -0.1476220000 -2.0046290000

H 4.4707540000 -1.3420690000 -2.2574380000

O 4.0631670000 -2.2556820000 -2.2818790000

H 3.0593290000 -2.2005020000 -2.2818880000

O 1.0293260000 -1.7265850000 -2.1917800000

H 0.8536520000 -2.3301600000 -1.4333940000

H 0.4878630000 -2.0477100000 -2.9165870000

H -2.3240700000 -2.0498940000 -0.8477470000

H -2.3128320000 -2.1201240000 0.7227460000

O -2.1198040000 -2.6303450000 -0.0893040000

H -0.6127560000 -3.0873790000 -0.1115160000

O 0.3716640000 -3.2916600000 -0.1154930000

H 0.5307220000 -4.2793360000 -0.1411640000

H -1.3593020000 3.4698450000 0.0528940000

O -2.1051950000 2.4371840000 0.0136260000

H -3.0653550000 2.5720510000 0.0060950000

O -4.9039610000 2.1933270000 0.0209550000

H -5.3755720000 1.8678210000 0.8383240000

H -5.3732500000 1.9111030000 -0.8136540000

H -2.3431120000 -0.1874080000 -2.2781490000

O -2.6712580000 -1.1022660000 -2.3048810000

H -3.6330170000 -1.0540750000 -2.3669160000

O -5.5888190000 -1.2431770000 -2.2688560000

H -5.9734070000 -1.6905220000 -1.4569010000

H -5.9711040000 -1.6473190000 -3.1042230000

C 0.6215150000 4.7154100000 0.0473350000

H 1.6574640000 2.9282380000 0.0789120000

N -0.4526030000 4.2449980000 0.0625480000

HNC···$(H_2O)_{20}$

H 5.0218320000 0.6010360000 1.3099030000

H 5.0228180000 0.7043260000 2.9428170000

O 5.2658820000 0.1342690000 2.1590630000

H -1.8380290000 1.9453370000 1.3450460000

H -1.8295400000 2.2816650000 2.8612030000

O -1.6833300000 1.5693640000 2.2356170000

H 0.1677080000 1.1137040000 2.2312420000

O 1.1106840000 0.9373730000 2.1084190000

H 1.1913520000 -0.0328660000 2.1632600000

H 4.4678260000 -1.3269280000 2.2519250000

O 4.0631970000 -2.2403450000 2.3097750000

H 3.0591850000 -2.1884960000 2.3070780000

O 1.0880520000 -1.8223370000 2.3297980000

H 0.6214590000 -2.1761880000 3.0888250000

H 0.8648350000 -2.4040270000 1.5766920000

H -2.3859300000 -0.1869750000 2.3684880000

O -2.6965950000 -1.1037830000 2.3405140000

H -3.6600930000 -1.0681630000 2.3766230000

O -5.5919850000 -1.2605170000 2.2528720000

H -5.9736640000 -1.6347710000 3.1022570000

H -5.9744820000 -1.7387310000 1.4576460000

H 5.0196990000 0.3235970000 -3.0772680000

H 5.0203970000 0.4268130000 -1.4443690000

O 5.2636760000 -0.1431620000 -2.2282010000

H 5.2077420000 2.4236420000 -0.1777200000

O 4.6664640000 1.6329150000 -0.1419470000

H 3.7284480000 1.9232070000 -0.1216490000

O 2.0317050000 2.1490550000 -0.0473750000

H 1.6932250000 1.7031330000 0.7770580000

H 1.4197160000 1.1500470000 -1.4743860000

H -1.5874100000 1.6987780000 -3.2766720000

H -1.9367350000 2.0976490000 -1.0464860000

O -1.5009920000 1.2271250000 -2.4434370000

H -0.5119870000 1.0197720000 -2.3627740000

O 0.9972110000 0.7043470000 -2.2299420000

H 1.1304140000 -0.2582970000 -2.1209760000

H 4.4655980000 -1.6043960000 -2.1353970000

O 4.0609500000 -2.5177990000 -2.0773410000

H 3.0569390000 -2.4659350000 -2.0801600000

O 1.0880210000 -2.0472770000 -2.1080630000

H 0.8724930000 -2.5515410000 -1.2985300000

H 0.5994000000 -2.4656790000 -2.8190590000

H -2.3151580000 -2.1769370000 -0.6592900000

H -2.3079950000 -2.0905830000 0.9039010000

O -2.1350820000 -2.6885980000 0.1523920000

H -0.6082920000 -3.1668560000 0.1684490000

O 0.3768370000 -3.3676650000 0.1806160000

H 0.5389890000 -4.3531950000 0.2428730000

H -1.1730920000 3.9447640000 -0.1682070000

O -2.2160560000 2.6190710000 -0.2550320000

H -3.1882990000 2.5569300000 -0.2483560000

O -4.9163560000 2.0912660000 -0.1618660000

H -5.3855210000 1.8386200000 0.6822340000

H -5.3863030000 1.7343200000 -0.9670120000

H -2.3454170000 -0.4561480000 -2.3227810000

O -2.6914540000 -1.3526200000 -2.1880360000

H -3.6522020000 -1.3016730000 -2.2529420000

O -5.5942620000 -1.5379450000 -2.1343870000

H -5.9758900000 -1.9122330000 -1.2849720000

H -5.9767250000 -2.0161870000 -2.9295870000

C 0.6214630000 5.1995220000 0.0347260000

H 1.7221450000 3.0608210000 -0.0025130000

N -0.3572530000 4.5670880000 -0.074612000

References

(1) Lane, J. R. CCSDTQ Optimized Geometry of Water Dimer. *J. Chem. Theory Comput.* **2013**, *9*, 316.
